# Review of Mix-and-Match Approach and Binocular Intraocular Lens Systems

**DOI:** 10.3390/jcm14124263

**Published:** 2025-06-16

**Authors:** Tadas Naujokaitis, Grzegorz Łabuz, Ramin Khoramnia, Gerd U. Auffarth

**Affiliations:** 1 The David J Apple Center for Vision Research, Department of Ophthalmology, University of Heidelberg, INF 400, 69120 Heidelberg, Germany; 2 Department of Ophthalmology, Faculty of Medicine and University Hospital Carl Gustav Carus, TU Dresden, Fetscherstr. 74, 01307 Dresden, Germany

**Keywords:** intraocular lens, IOL, mix-and-match, combination, IOL systems

## Abstract

In the mix-and-match approach and in binocular intraocular lens (IOL) systems, two different IOL models are implanted in each eye to achieve the desired binocular outcome. In the mix-and-match approach, the surgeon selects the IOL models to be combined according to the clinical situation, the patient’s needs, and personal preference. Combinations described in the literature include, among others, two bifocal IOLs, an extended-depth-of-focus (EDoF) IOL with a bifocal lens, a trifocal lens with an EDoF IOL or an enhanced monofocal IOL, and two EDoF models utilizing different optical principles. The outcomes depend on the selected combination of IOL models. Binocular IOL systems consist of a fixed combination of two lenses, developed to be complementary in binocular vision. Initial data indicate that they can achieve a depth of focus similar to that with a bilateral implantation of a trifocal IOL. However, comparative studies are needed to evaluate if the postoperative binocular outcome differs from that achieved with the conventional approach. The mix-and-match implantation and binocular IOL systems expand the options available to tailor the IOL selection to the patient’s needs.

## 1. Introduction

The wide range and increasing complexity of intraocular lens (IOL) models available pose a challenge in selecting the optimal IOL model for the patient. Each IOL type has its own benefits and drawbacks, and the personalized IOL selection involves matching the specific patient needs to the properties of the IOL. Traditionally, the same model IOL is implanted in both eyes to provide a similar visual impression with each eye. The mix-and-match approach and binocular IOL systems contrast with the traditional approach by using a combination of two different IOL models. The goal of these approaches is an optimal binocular outcome, which depends on the optical properties of the two IOL models. This review covers both the non-standardized mix-and-match technique, used by physicians to combine two IOL models of their preference, and the commercial binocular IOL systems, which can be seen as an attempt to further advance and standardize the mix-and-match concept [1,2].

## 2. Literature Search

The literature search was performed using the search terms “mix-and-match”, “mix and match”, “IOL combination”, “IOL system”, “Artis Symbiose”, and “WELL Fusion” in the PubMed database and using the Google Scholar search engine on 9 April 2025.

## 3. Mix-and-Match Approach

The concept of combining two different IOL models which were originally designed for a binocular implantation of the same model IOL is called a mix-and-match approach. This is a non-standardized technique, and each of the two IOL models can be individually selected by the physician. On the one hand, this allows for a highly personalized approach, tailoring the IOL model selection to the needs of the patient [3]. On the other hand, a good understanding of the particular clinical situation and knowledge of the optical principles of different IOL models are required in order to avoid mistakes and achieve a good outcome [3]. The selected IOLs can share some optical characteristics, such as the diffractive technology, or use completely different optical principles [3]. Even though numerous studies analyzed the outcomes with the mix-and-match technique, each IOL combination can lead to different outcomes and the variety of combinations used complicates the comparison of published results. This review aims to cover the most common IOL combinations used in a mix-and-match approach and summarize the published data on their performance.

### 3.1. Combination of Two Bifocal IOLs with High Add Powers

**Figure 1:**
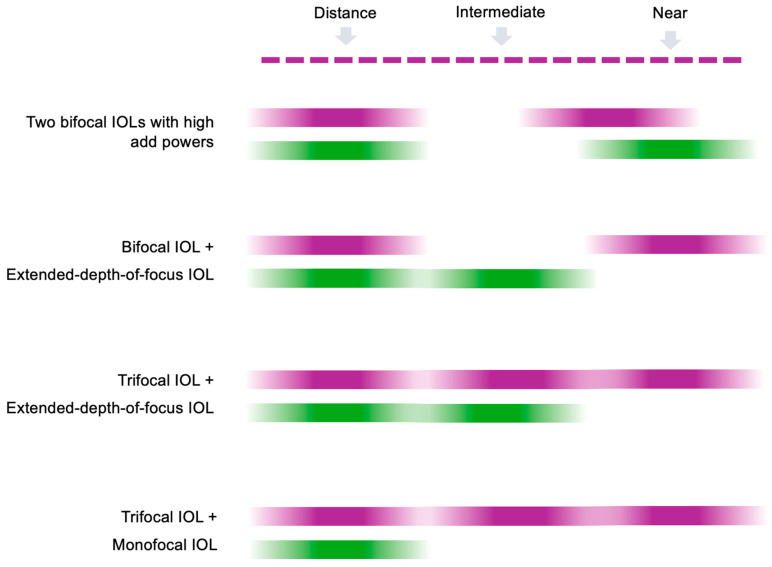
A simplified illustration of different mix-and-match approaches described in the text. Depth of focus ranges of individual intraocular lens (IOL) models are shown, which are combined in binocular vision. The actual depth of focus varies depending on the particular IOL model and may differ from that presented in this figure.

One of the main motivations in the mix-and-match approach is the increased depth of focus, associated with a higher spectacle independence [3]. By using this technique, the binocular depth of focus can be expanded when compared to a bilateral implantation of the same model IOL [4,5]. One of the possible IOL combinations to achieve a greater depth of focus is the implantation of two bifocal IOLs with different add powers (Figure 1) [4,5,6,7]. In such an approach, both eyes have good distance vision as emmetropia is targeted for both IOLs, while different add powers complement each other, resulting in overlapping monocular defocus curves [4,5,6,7]. Although the outcomes achieved vary between studies, and there is a lack of data on direct comparisons of mix-match-approaches, the depth of focus achieved with such a system should theoretically depend on the difference in the add powers [4,5,6,7]. A larger difference between the add powers should theoretically result in a boarder depth of field but could come at the expense of binocular near vision, similar to what is observed with monovision treatments [8,9].

Jiang et al. compared a mix-and-match implantation of the diffractive bifocal Acrysof IQ ReSTOR +2.5 D (Alcon, Fort Worth, TX, USA) and Acrysof IQ ReSTOR +3.0 D (Alcon) IOLs with a bilateral implantation of the Acrysof IQ ReSTOR +3.0 D IOL and reported better binocular uncorrected intermediate visual acuity (UIVA) with the mix-and-match approach even though the add power of the IOLs differed by only 0.5 D [4]. Lee et al. presented the outcomes after the implantation of two diffractive bifocal IOLs, ZKB00 (Johnson & Johnson Vision, Santa Ana, CA, USA) and ZMB00 (Johnson & Johnson Vision), with +2.75 D and +4.00 D add powers [6]. The mean ± standard deviation (SD) postoperative binocular uncorrected distance visual acuity (UDVA) was 0.01 ± 0.04 logarithm of the minimum angle of resolution (logMAR), UIVA was 0.16 ± 0.05 logMAR, and uncorrected near visual acuity (UNVA) was 0.11 ± 0.07 logMAR [6].

Although the mix-and-match approach using two bifocal IOLs with different add powers may produce a wider depth of focus than a bilateral implantation of a bifocal IOL with the same add power or a bilateral implantation of an EDoF IOL, the mix-and-match approach seems to result in similar or worse outcomes in comparison to a bilateral trifocal IOL implantation [7,10,11]. A prospective study by Paik et al. compared the results after a mix-and-match implantation of diffractive bifocal IOLs ZKB00 with an add power of +2.75 D in the dominant eye and ZLB00 (Johnson & Johnson Vision) with an add power of +3.25 D) in the fellow eye with those after a bilateral diffractive extended-depth-of-focus (EDoF) IOL TECNIS Symfony (Johnson & Johnson Vision) and a bilateral diffractive trifocal IOL FineVision PodFT (BVI, Waltham, MA, USA) implantation [7]. In all three groups, the visual acuity at a far distance was comparable [7]. In the binocular defocus curves, the mix-and-match approach provided similar results to those after bilateral trifocal IOL implantation, outperforming the EDoF lens at a defocus of −2.5 D, which corresponds to a viewing distance of 40 cm [7]. A prospective comparative study reported a mix-and-match implantation of bifocal IOLs ReSTOR +2.50 D/+3.00 D resulted in worse intermediate and near vision than a bilateral implantation of the trifocal AcrySof IQ PanOptix (Alcon) IOL [10]. Similarly, another study compared the mix-and-match approach using the same bifocal IOL models with a bilateral implantation of the FineVision (BVI) trifocal IOL and also found better intermediate and near binocular visual acuities with the trifocal lens [11]. Therefore, it can be summarized that the mix-and-match approach combining two bifocal lenses with high add powers (+2.50 D and higher) does not seem to provide an advantage against trifocal IOLs.

### 3.2. Combination of Bifocal IOL with Extended-Depth-of-Focus IOL

The other possible mix-and-match strategy is a combination of EDoF and bifocal IOLs [12,13,14,15]. It should be clarified that the EDoF category is heterogenic and differences in terminology exist [16,17,18,19]. Some of the lenses which are called EDoF truly elongate the depth of focus through the use of small-aperture or aberration-based designs, while others are bifocal lenses with a low addition [16,17,18,19]. For the purpose of this review, the lenses that are designed to primarily provide the distance and intermediate vision are called EDoF, as the monocularly covered ranges are among the most relevant characteristics when combining lenses in the mix-and-match approach.

Liu et al. compared the outcomes after a bilateral implantation of the bifocal LENTIS Mplus LS-313MF30 (Teleon Surgical, Spankeren, The Netherlands) and the mix-and-match approach with the refractive low-add-power (+1.5 D) bifocal IOL LENTIS Mplus LS-313MF15 (Teleon Surgical) implanted in the dominant eye and the refractive bifocal LENTIS Mplus LS-313MF30 with a higher add power of +3.0 D in the fellow eye [5]. The mix-and-match approach provided a better intermediate vision (mean ± SD UIVA of 0.10 ± 0.12 logMAR vs. 0.23 ± 0.14 logMAR) and a higher spectacle independence at intermediate distance (92.0 % vs. 59.26 %) than the bilateral implantation of the bifocal Mplus LS-313MF30 lens [5].

Lee et al. presented the results after the implantation of a Tecnis Symfony (Johnson & Johnson Vision) EDoF IOL in one eye and a ZLB00 bifocal IOL in the fellow eye [13]. While they found that 81.1% of patients were “more than satisfied” with their near vision, 21.6% of patients complained of severe glare and halo [13]. Spectacle independence for near vision was achieved in 91.9% of patients [13]. 

The mix-and-match approach combining the Tecnis Symfony EDoF IOL with the ZMB00 or ZLB00 bifocal IOLs was compared with a bilateral implantation of the trifocal AcrySof IQ PanOptix IOL by Song et al. and Medeiros et al. [20,21]. In one study, the mix-and-match approach provided a slightly better binocular VA throughout most of the defocus range studied, while the other study only found differences between −0.50 D and −1.00 D, where the mix-and-match outperformed the trifocal IOL, and at −4.0 D, where the trifocal group had a better VA [1,20,21]. Based on the results of these studies, it appears that a combination of an EDoF lens with a bifocal lens of a higher add power can result in comparable or even slightly better outcomes than in a bilateral trifocal IOL implantation, especially at intermediate distances.

### 3.3. Combination of Trifocal IOL with Extended-Depth-of-Focus or Monofocal IOL

It was also attempted to combine a trifocal lens with an EDoF or enhanced monofocal IOL [22,23,24,25,26]. While outcomes vary depending on the exact IOL models used, Kim et al. reported lower rates of photic phenomena but worse near vision with these mix-and-match approaches, when compared to a bilateral trifocal IOL implantation [24]. It should be noted that a combination of a trifocal and a monofocal IOL can lead to considerable disparity in visual impression between the eyes. From our experience, they may not be well tolerated by some patients and could, in rare cases, necessitate IOL exchange [27]. In general, it seems safer to avoid multifocal IOL implantation in patients who are unwilling to accept photic phenomena than trying mix-and-match strategies to reduce photic phenomena.

### 3.4. Mix-and-Match Strategies in Complex Cases

Finally, as a highly personalized approach, the mix-and-match strategy can be useful in complex cases [3]. Patients with irregular astigmatism, for example, can benefit from the implantation of the small-aperture IC-8 IOL (Bausch + Lomb, Bridgewater, NJ, USA), which utilizes the pinhole effect to reduce the influence of corneal higher-order aberrations on the image quality [3,28,29]. If the fellow eye has no irregular astigmatism, a conventional, preferably refractive EDoF lens can be implanted in that eye to match the defocus characteristics of the IC-8 [28]. Baur et al. published a case report where a patient received the IC-8 IOL in the eye with an irregular astigmatism after corneal refractive surgery, and an EDoF IOL AcrySof IQ Vivity (Alcon) in the fellow eye which had no irregular astigmatism [28]. The patient achieved a binocular uncorrected vision of approximately 20/20 at far and intermediate distances and approximately 20/32 at near [28]. The small-aperture IOL can also be combined with a monofocal IOL [29,30]. The IC-8 IOL is often implanted unilaterally out of concerns of mesopic and scotopic vision impairment due to the reduction in incoming light, although good results were also reported after its bilateral implantation [3,28,29]. The small-aperture lens can also be used in patients with healthy corneas, in order to extend their depth of focus [31]. Son et al. reported outcomes of the IC-8 IOL implanted in one eye and the refractive Lentis Mplus LS-313 MF20 IOL in the fellow eye, with a binocular UDVA and UIVA of approximately 20/20, and a UNVA of 20/25 [31]. Only minimal photic phenomena were reported by the patients [31]. The patients reported a mean halo size of 32.54 ± 22.38 (out of 100) and a mean halo intensity of 34.46 ± 21.95 (out of 100), a mean glare size of 9.00 ± 17.47 (out of 100) and a mean glare intensity of 9.92 ± 16.84 (out of 100) [31]. 

## 4. Binocular IOL Systems

Similarly to the mix-and-match approach, the commercial binocular complementary IOL systems consist of two different IOL models. However, these IOLs are specifically designed to be complementary in binocular implantation [1,3]. Therefore, the binocular IOL systems can be seen as a development of a non-standardized mix-and-match approach into a commercially available system [1].

### 4.1. Artis Symbiose

The binocular IOL system Artis Symbiose (Cristalens Industrie, Lannion, France) consists of two aspheric hybrid multifocal-EDoF lenses, MID and PLUS, made of hydrophobic acrylic material [32]. Their central zone of 4.2 mm features a diffractive pattern and the peripheral part of the optic is refractive only [32]. The lenses are designed to be complementary in binocular implantation: the MID lens is optimized for the intermediate range and the PLUS lens is designed to provide a superior vision at a near distance, while they both equally contribute to distance vision [1,3]. This is achieved by a combination of the far focus, produced by the zeroth diffractive order, and an EDoF zone, created by multiple merging additions produced by the first diffractive order [1,32]. This EDoF zone is used to provide the intermediate vision with MID and the near vision with PLUS [32]. In binocular implantation, their depth of focus covers far, intermediate, and near distances, creating a result similar to that with a trifocal IOL [1,32]. 

**Figure 2:**
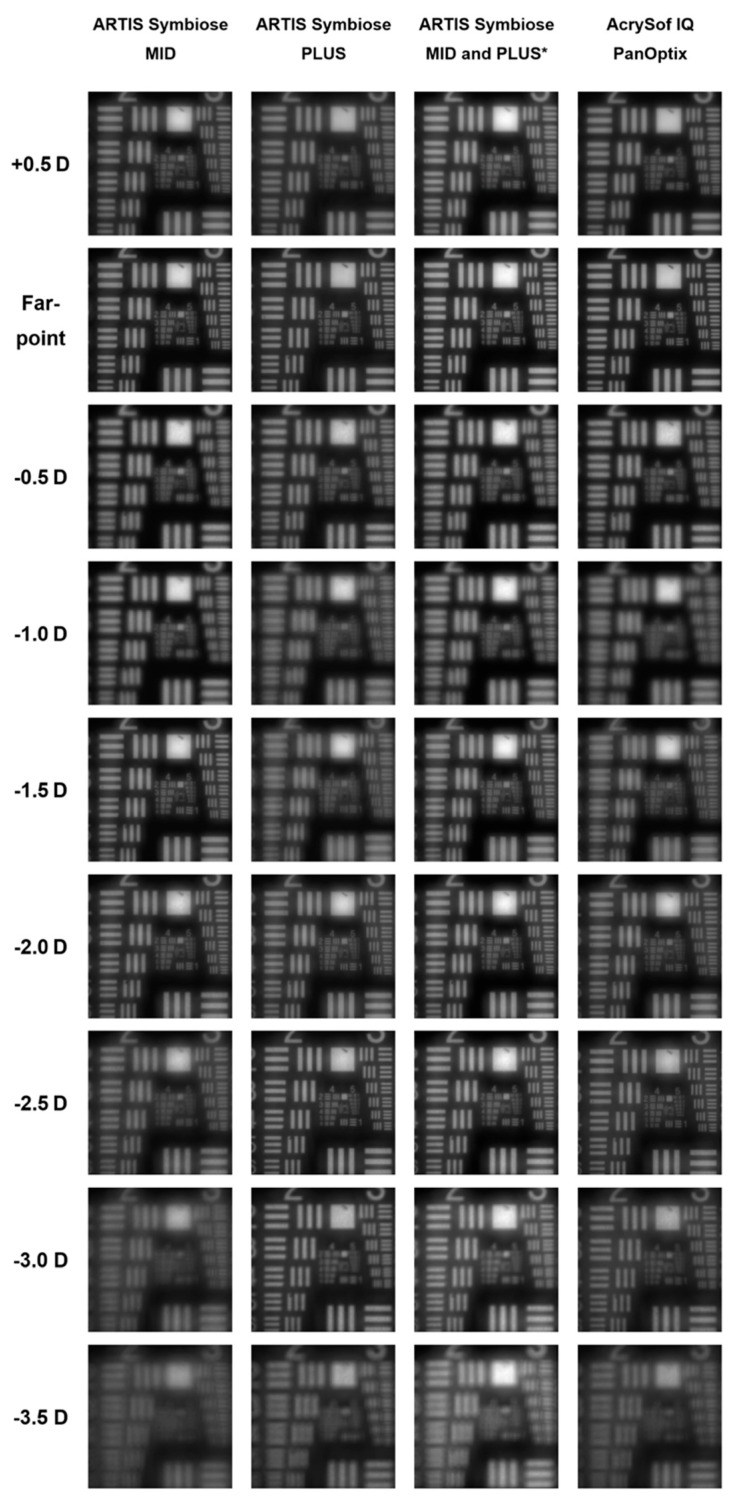
United States Air Force resolution chart photographed through the MID and PLUS lenses of the Artis Symbiose binocular IOL system and through the trifocal IOL AcrySof IQ PanOptix using an optical bench setup. * —Simulation of binocular vision with the binocular IOL system, obtained by applying a quadratic summation of the MID’s and PLUS’s images. Reproduced from Naujokaitis et al. [1]. [CCBY4.0].

In an optical-bench-based study which evaluated the total depth of focus using monochromatic light and a single frequency of the modulation transfer function, the simulated combined implantation of MID and PLUS resulted in a greater depth of focus than that of trifocal lenses, even though each of the MID and PLUS lenses separately had a lower depth of focus compared to a trifocal IOL [33]. A later laboratory study performed simulations of visual acuity using polychromatic light and multifrequency metrics aiming to more closely match real-world situations [1]. Its results suggested that the binocular IOL system could theoretically provide a better intermediate vision and a more extended depth of focus as the simulated defocus curve of the MID outperformed the trifocal AcrySof IQ PanOptix IOL in the intermediate range, while the PLUS provided a slightly more extended near focus (Figure 2) [1]. However, the performance of the MID lens was worse in the near range and the PLUS had lower simulated VA in the intermediate range, and it remained unclear how these differences between the lenses affect binocular vision [1]. Therefore, the study acknowledged the limitations of the optical-bench studies in predicting binocular visual acuity [1].

Clinical data with the Artis Symbiose binocular IOL system were reported by Lajara-Blesa et al. [32]. Their prospective non-comparative study found a binocular visual acuity of 20/20 or better in the range from +0.5 D to −2.5 D of defocus [32]. The shapes of the monocular defocus curves resembled those in the laboratory study [1,32]. Both lenses provided a similar visual acuity at 0 D of defocus, which corresponds to the distance vision, the MID outperformed the PLUS at defocus levels corresponding to the intermediate distances, and the PLUS outperformed the MID at high defocus corresponding to near, demonstrating their complementary design [32]. A study by McNeely et al. also analyzed the outcomes with Artis Symbiose and found a mean ±SD binocular UDVA of −0.07 ± 0.06 logMAR, a UIVA of 0.03 ± 0.10 logMAR and a UNVA of 0.07 ± 0.08 logMAR [2]. The clinical binocular defocus curve in the study by McNeely et al. had nearly identical values at no defocus to those reported by Lajara-Blesa et al. but worse (up to one line difference) values at higher myopic defocus [2,32]. While the monocular performance of the MID lens matched that reported by Lajara-Blesa et al., the PLUS lens had a worse visual acuity at −2.5 D and higher myopic defocus, probably leading to the difference in binocular visual acuity between the studies [2,32]. A randomized controlled trial attempted to compare the Artis Symbiose IOL system to a bilateral diffractive EDoF lens AT LARA (Carl Zeiss Meditec, Jena, Germany) implantation but was limited by a small sample size of 17 patients with postoperative data in both groups in total [34]. The shape of the binocular defocus curve with Artis Symbiose differed considerably from that in the previously discussed studies and resembled the shape of the defocus curve with the EDoF IOL [34]. The mean visual acuity deteriorated monotonously with increasing defocus and was only approximately 0.25 logMAR at −2.5 D [34]. The small sample size and high inter-subject variability, with the SD at higher defocus values exceeding 0.20 logMAR, are possible reasons for the low agreement of the results of this study with those from other studies [34]. 

### 4.2. WELL Fusion

The WELL Fusion (SIFI, Catania, Italy) is another commercial binocular IOL system, consisting of two refractive lenses using zones with a spherical aberration of opposite signs to extend the depth of focus [35]. The IOLs are manufactured from a hydrophilic acrylic material with a hydrophobic surface [35]. The Mini WELL IOL is optimized for far and intermediate distances, while the Mini WELL PROXA lens primarily covers far and near distances, thus resembling the MID and PLUS lenses in the Artis Symbiose IOL system [35]. Clinical data for the WELL Fusion IOL system are limited to a prospective non-comparative study by Mastropasqua et al., which included 30 patients [35]. The monocular defocus curve of the Mini WELL IOL had visual acuity peaks at no defocus and −1.5 D of defocus, while the performance of the Mini WELL PROXA peaked at no defocus and −1.5 D to −2.0 D [35]. A mean binocular visual acuity of 20/25 or better was achieved in the range from +0.5 D to −2.5 D of defocus, resembling the outcomes reported by McNeely et al. with Artis Symbiose [2,35]. However, differently from the studies reporting the results with Artis Symbiose, the mean binocular visual acuity at −1.0 D, −1.5 D, and −3.0 D of defocus dropped below the mean monocular visual acuity curves of individual IOLs, and future studies are needed to evaluate the binocular summation with both IOL systems [35]. In general, the visual acuity outcomes were favorable, and the mean (±SD) binocular UDVA of 0.03 ± 0.11 logMAR, UIVA of 0.05 ± 0.10 logMAR, and UNVA of −0.03 ± 0.08 logMAR were achieved at 3 months post-surgery, comparable to visual acuity outcomes with a trifocal IOL [3,35]. Clinical studies reporting outcomes with binocular IOL systems are summarized in Table 1.

## 5. Considerations When Implanting Two Different IOL Models

### 5.1. Tolerance to Differences in Visual Impression Between the Eyes

One of the potential issues that arises when implanting two different IOL models in each eye is the possibility that the different visual impressions with each eye may not be well-tolerated. Studies reporting outcomes with pseudophakic monovision, which also results in considerable differences in visual impression between the eyes, found varying patient satisfaction [9,36]. While most patients could tolerate the difference, the satisfaction rate seemed to be higher when there was a lower offset between the refractive targets [1,8,9,37]. The inter-ocular differences in visual impressions with the mix-and-match approach depend on the IOL combination used. For example, a combination of two bifocal IOLs which both use the same optical technology to achieve multifocality and only slightly differ in their add powers is unlikely to be less tolerated than a bilateral implantation of a bifocal lens. However, an implantation of markedly different IOLs, such as a combination of a multifocal IOL in one eye and a monofocal IOL in the fellow eye, could be more problematic in some patients [38]. The binocular IOL systems are designed using a matching optical technology and only moderate differences between the lenses, making them unlikely to cause intolerance due to different visual impressions between the eyes. Still, it is recommended to inform the patients that the goal of the surgery is an optimal binocular and not monocular vision [38].

### 5.2. Differences in Contrast Perception

Differences between IOLs could lead to one eye perceiving a higher image contrast than the fellow eye. Not only can this difference between images in rare cases be perceived as disturbing, as discussed above, but the eye with the lower perceived contrast could theoretically decrease the overall binocular contrast perception [39]. The rule of quadratic summation, proposed by Legge and Rubin, indicates that the eye which sees a higher contrast dominates the binocular suprathreshold contrast perception, but a reduction in monocular contrast in the eye with a lower contrast still slightly reduces the binocular contrast [39]. A slight difference in perceived image contrast between the eyes is unlikely to markedly affect the perception of binocular image contrast. In IOL combinations where the models differ considerably, the reduction in binocular contrast perception might be more pronounced. However, the clinical relevance of differences in suprathreshold contrast perception in patients implanted with mix-and-match and binocular IOL systems remains unclear.

### 5.3. Stereopsis

Another potential issue caused by differing images between the eyes is impaired stereopsis [40]. This side effect is well-studied in contact lens wearers with monovision [41]. A review by Bohac et al. found that a reduction in stereoacuity occurs in unilateral multifocal IOL implantation, while binocularly implanted patients have stereoacuity values in the normal range [40]. To achieve a high stereoacuity, the image perceived by both eyes has to be of sufficient quality, which can be impaired in a mix-and-match implantation of two highly different IOLs. However, moderate differences between lenses in mix-and-match implantation and binocular intraocular lens systems are unlikely to cause issues. In the study by Lajara-Blesa et al., patients with the Artis Symbiose IOL system were found to achieve a median (interquartile range) stereoacuity of 40.0 (12.5) seconds of arc, which was the lowest value on the Titmus test used by the examiners [32]. A study by McNeely et al., which assessed outcomes with Artis Symbiose after refractive lens exchange, used the TNO stereotest to measure stereoacuity and reported that the patients maintained or improved in stereoacuity postoperatively [2]. All patients achieved at least gross stereoacuity, 90.1% had a stereoacuity of 480 s of arc or better, and 50% achieved 120 s of arc or better [2]. The authors concluded that the implantation of the complementary MID and PLUS IOLs did not negatively affect the stereopsis [2].

### 5.4. Binocular Summation

Despite the importance of binocular vision for the functioning of the mix-and-match concept and binocular IOL systems, binocular summation in cases where two different IOL models are implanted remains to be investigated. The scientific evidence of binocular summation with multifocal lenses is limited to the analysis of binocular summation in the bilateral implantation of the same-model lens [42]. The mean binocular summation was reported to be approximately 0.07 logMAR to 0.10 logMAR, when compared to the monocular visual acuity in the eye with a better visual acuity, after a bilateral implantation of a bifocal IOL [42]. A study of binocular summation in phakic subjects found the highest amount of binocular summation when both eyes perceived similar images and the binocular summation decreased with increasing contrast disparity between the eyes [43]. This could potentially negatively affect the binocular summation with mix-and-match and binocular IOL systems. However, it should be noted that the amount of binocular summation appears to be lower at high visual acuity levels, which could be associated with the number of neurons activated [44]. In many cases with the mix-and-match approach and binocular IOL systems, a relatively good monocular visual acuity is maintained throughout a wide range of defocus. At these high visual acuity levels, the low amount of binocular summation that can potentially occur might diminish the clinical relevance of differences in binocular summation between approaches.

### 5.5. Practical Considerations

High patient expectations for spectacle independence and the demand for approaches tailored to suit individual needs motivate the search for personalized IOL strategies. Patients expect the surgeon to customize IOL selection to accommodate the individual lifestyle requirements. Both mix-and-match approach and binocular IOL systems are valuable tools which expand the IOL options available and allow the surgeons to provide a higher degree of personalization for their patients. While the complexity of the surgeries themselves, when implanting two different IOL models in each eye, is similar to that when implanting the same-model lens in both eyes, the use of different models add to the complexity of the surgical planning and care is needed to avoid errors. Furthermore, in case of intraoperative complications when a safe implantation in the capsular bag is no longer possible, the use of a different IOL model than initially planned can be necessary. As a mix-and-match approach relies on two IOLs specifically selected to match and complement each other, an unplanned use of another IOL model might prevent achieving the optimal binocular outcome, and patients should be informed preoperatively about the possibility of such event.

### 5.6. Directions for Future Research

Binocular IOL systems were introduced relatively recently, and more research is needed to evaluate their performance in comparison to conventional approaches. In addition, future studies are needed to compare the outcomes with mini-monovision using EDoF and enhanced monofocal IOLs with those achieved with binocular IOL systems and the mix-and-match approach. Comparative IOL studies, particularly in the form of randomized controlled trials, are desirable to enable a direct comparison of binocular outcomes achieved with different approaches.

## 6. Conclusions

In conclusion, mix-and-match approach and binocular IOL systems expand the options available in order to achieve an outcome that is tailored to the patient’s needs. While the mix-and-match approach may increase the complexity of preoperative planning, it can provide flexibility in matching the IOL characteristics to the particular clinical situation. The outcomes with the mix-and-match approach depend on the combination of IOL models used and therefore the knowledge of the optical principles of IOLs is required to select the combination that is suitable for the particular patient. Binocular IOL systems simplify and further advance the mix-and-match approach by providing a fixed combination of two IOLs specifically designed to complement each other in binocular vision. While their initial results are promising and suggest similar outcomes to those after a bilateral trifocal IOL implantation, comparative studies are needed to evaluate their benefits and drawbacks against the traditional approach.

## Figures and Tables

**Table 1:** Clinical studies reporting outcomes with binocular intraocular lens (IOL) systems.

Study (Year of Publication)	Number of Patients	Binocular Visual Acuity	Binocular Defocus Curve	Spectacle Independence	Photic Phenomena
Artis Symbiose					
Lajara-Blesa et al. (2023) [32]	23 (6-month data)	6 months post-surgery: median (interquartile range) UDVA −0.10 (0.10) logMAR; Mean ± standard deviation CDVA −0.11 ± 0.10 logMAR; UIVA (90 cm) −0.03 ± 0.20 logMAR; DCIVA (90 cm) 0.05 ± 0.12 logMAR; UIVA (70 cm) 0.03 ± 0.11 logMAR; DCIVA (70 cm) 0.00 ± 0.09 logMAR; UNVA 0.06 ± 0.10 logMAR; DCNVA 0.04 ± 0.09 logMAR	0.20 logMAR and better between −3.0 D and +1.0 D; 0.10 logMAR and better −3.0 D and +0.5 D	Not reported	Light Distortion Analyzer: Monocular MID lens: 12.57 (6.61) %, Monocular PLUS lens: 14.99 ± 5.70%, Binocular: 10.36 ± 4.42%
McNeely et al. (2023) [2]	44	12 months post-surgery: UDVA −0.07 ± 0.06 logMAR UIVA 0.03 ± 0.10 logMAR UNVA 0.07 ± 0.08 logMAR	0.20 logMAR and better between −3.0 D and +1.0 D; 0.10 logMAR and better −2.5 D and +0.5 D	93.2% complete spectacle independence	Halo: 0.36 ± 0.53 (scale 0 to 3) Glare: 0.39 ± 0.62 (scale 0 to 3)
Ivellio-Vellin et al. (2024) [34]	14 initially included in the Artis Symbiose group (28 patients in total), 11 excluded or lost to follow-up	6 months post-surgery median (mean, range): UDVA 0.0 (0.0, −0.1 to 0.0) logMAR; CDVA −0.1 (−0.1, −0.1 to 0.0) logMAR; UIVA 0.1 (0.1, −0.1 to 0.3) logMAR; DCIVA 0.1 (0.1, −0.1 to 0.3) logMAR; UNVA 0.1 (0.1, 0.0 to 0.4) logMAR; DCNVA 0.1 (0.1, 0.0 to 0.5) logMAR	0.20 logMAR and better between −2.0 D and +1.0 D; 0.10 logMAR and better −1.25 D and +0.5 D	Not reported	Binocular halo size: approx. 1.0°
WELL Fusion					
Mastropasqua et al. (2023) [35]	30	3 months post-surgery: UDVA 0.03 ± 0.11 logMAR; CDVA −0.02 ± 0.08 logMAR; UIVA 0.05 ± 0.10 logMAR; DCIVA 0.06 ± 0.09 logMAR; UNVA (40 cm) −0.03 ± 0.08 logMAR; DCNVA (40 cm) −0.05 ± 0.05 logMAR; UNVA (33 cm) 0.06 ± 0.08 logMAR; DCNVA (33 cm) 0.05 ± 0.06 logMAR	0.20 logMAR and better between −3.5 D and +1.0 D; 0.10 logMAR and better −2.5 D and +0.5 D	National Eye Institute Refractive Error Quality-of-Life instrument-42 Dependence on correction: approx. 90–93 out of 100	National Eye Institute Refractive Error Quality-of-Life instrument-42 Glare: approx. 70–76 out of 100
